# Endogenous Enzymatic Activity of Primary and Permanent Dentine

**DOI:** 10.3390/ma14144043

**Published:** 2021-07-20

**Authors:** Tatjana Maravic, Lorenzo Breschi, Federica Paganelli, Giulio Alessandri Bonetti, Stefano Martina, Gianni Di Giorgio, Maurizio Bossù, Antonella Polimeni, Vittorio Checchi, Luigi Generali, Franklin R Tay, Milena Cadenaro, Annalisa Mazzoni

**Affiliations:** 1 Department of Biomedical and Neuromotor Sciences, University of Bologna-Alma Mater Studiorum, 40125 Bologna, Italy; tatjana.maravic2@unibo.it (T.M.); paganellifederica73@gmail.com (F.P.); giulio.alessandri@unibo.it (G.A.B.); annalisa.mazzoni@unibo.it (A.M.); 2 Department of Medicine, Surgery and Dentistry, University of Salerno, 84081 Baronissi (Salerno), Italy; smartina@unisa.it; 3 Department of Oral and Maxillofacial Science, “Sapienza” University of Rome, 00161 Rome, Italy; gianni.digiorgio@uniroma1.it (G.D.G.); maurizio.bossu@uniroma1.it (M.B.); antonella.polimeni@uniroma1.it (A.P.); 4 Department of Surgery, Medicine, Dentistry and Morphological Sciences with Transplant Surgery, Oncology and Regenerative Medicine Relevance, University of Modena and Reggio Emilia, 41124 Modena, Italy; vittorio.checchi@unimore.it (V.C.); luigi.generali@unimore.it (L.G.); 5 The Dental College of Georgia, Augusta University, Augusta, GA 30912, USA; ftay@augusta.edu; 6 Department of Medical Sciences, University of Trieste, 34125 Trieste, Italy; mcadenaro@units.it; 7 Institute for Maternal and Child Health-IRCCS “Burlo Garofolo”, 34137 Trieste, Italy

**Keywords:** MMPs, primary dentine, permanent dentine, zymography

## Abstract

Matrix metalloproteinases (MMPs) play an important role in tooth development and influence caries development and hybrid layer degradation. Literature is scant on the differences in the activity of MMPs between primary and permanent dentine. Accordingly, the aim of the present study was to investigate endogenous gelatinolytic activity in primary and permanent dentine. Separate batches of dentine powder were obtained from intact human primary and permanent molars (*n* = 6). Each batch was divided in two subgroups: (1) mineralised; and (2) demineralised with 10% H_3_PO_4_. After protein extraction, gelatine zymography was performed. Furthermore, in situ zymography was performed on dentine sections of the same groups (*n* = 3). The slices were polished, covered with fluorescein-conjugated gelatine and evaluated using a confocal microscope. In situ zymography data were analysed using two-way analysis of variance and post hoc Holm–Šidák statistics (α = 0.05). Primary dentine showed poorly defined bands in the zymograms that vaguely corresponded to the pro-form and active form of MMP-2 and the pro-form of MMP-9. In permanent dentine, demineralised powder demonstrated stronger gelatinolytic activity than mineralised powder. In situ zymography identified stronger enzymatic activity in primary etched dentine (*p* < 0.05). Stronger enzymatic activity recorded in primary dentine may be related to the differences in morphology and composition between primary and permanent dentine.

## 1. Introduction

The composition and structure of dentine vary in different parts of the tooth because of the presence of dentinal tubules. Overall, dentine is composed of approximately 70 weight % (45 volume %) mineral component and 20 weight % (33 volume %) organic matter, with the remaining fraction occupied by water [1,2]. The properties of dentine vary, depending on the part of the tooth where the dentine is located, the proximity of the pulp, and the influence of physiologic/pathologic processes such as aging, caries or sclerosis [1]. Furthermore, primary and permanent teeth differ in micromechanical and structural properties. Primary teeth have lower mineral content and less intertubular dentine, which probably accounts for their lower mechanical properties [3,4,5,6]. Bovine primary dentine was also found to be more susceptible to osteoclastic degradation [7]. Although primary and permanent dentine have a similar organic matrix [5], the primary dentine matrix seems to be more prone to endogenous enzymatic degradation [8], which could clinically underlie higher susceptibility towards caries and hybrid layer degradation.

There are limited data on the biochemical properties of primary dentine. Matrix-metalloproteinases (MMPs) are zinc and calcium ion-dependent endogenous dentinal proteases. They play important roles in tooth development but can influence caries progression and impair the longevity of dentine bonding by degrading the resin-sparse, water and exposed collagen-rich zones of the hybrid layers created by etch-and-rinse adhesives [9,10,11]. Hence, there is a need to understand the predisposition of endogenous dentinal enzyme activities in primary dentine and compare the results with activities present in permanent dentine. Although gelatine zymography in dentine powder and the in situ zymography of dentine sections have been used previously to examine the enzymatic activity in permanent dentine, they have not been used in primary dentine.

Accordingly, the objectives of the present study were to investigate the differences in the gelatinolytic activities of MMP-2 and MMP-9 in primary dentine and permanent dentine, and to determine whether gelatine zymography of dentine powder and in situ zymography of dentine thin sections are suitable for examining the endogenous enzymatic activities of primary dentine. The null hypothesis tested was that there are no differences in the endogenous gelatinolytic activities between primary and permanent dentine.

## 2. Materials and Methods

### 2.1. Gelatine Zymography

Gelatine zymography was performed in accordance with the method reported by Mazzoni et al. [12]. Mineralised dentine powder was obtained from 6 human third molars and 6 human primary molars. Dentine powder was obtained by freezing sectioned dentine blocks that were devoid of enamel and cementum in liquid nitrogen and triturating the blocks with a benchtop mill designed for the cryogenic grinding of small samples (MM400, Retsch GmbH, Haan, Germany). Aliquots of mineralised dentine powder were divided into 4 groups: G1—mineralised permanent dentine (control); G2—mineralised primary dentine; G3—demineralised permanent dentine (demineralised with 10 wt.% phosphoric acid (H_3_PO_4_) for 10 min at 4 °C; and G4—demineralised primary dentine (demineralised in the same manner as G3). All the groups were tested in duplicate.

After the etching procedure in G3 and G4, H_3_PO_4_ was neutralised using 5 M NaOH, centrifuged for 20 min at 4 °C (20,800× *g*), rinsed twice with water, and re-centrifuged at 4 °C. For protein extraction, dentine powder aliquots (2/group) were re-suspended in extraction buffer (50 mM Tris-HCl containing 5 mM CaCl_2_, 100 mM NaCl, 0.1% Triton X-100, 0.1% nonionic detergent P-40, 0.1 mM ZnCl_2_, 0.02% NaN_3_; pH = 6) for 24 h at 4 °C, sonicated for 10 min, and centrifuged for 20 min at 4 °C. The supernatant was removed and re-centrifuged. The protein content was concentrated in a centrifugal concentrator with 10,000 KDa cut-off (Vivaspin Sartorius Stedim Biotech, Goettingen, Germany) for 30 min at 4 °C (15,000 *g*, 3 times). The total protein concentration of the dentine extracts was determined using Bradford assay (Bio-Rad, Hercules, CA, USA). Dentine protein aliquots (60 μg) were diluted in Laemmli sample buffer at a 4:1 ratio and subjected to electrophoresis under non-reducing conditions in 10% sodium dodecyl sulphate–polyacrylamide gel electrophoresis (SDS-PAGE) containing 1 mg/mL of fluorescently labelled gelatine. Pre-stained, low-molecular-weight SDS-PAGE standards (Bio-Rad) were used as molecular-weight markers. The zymography was performed in triplicates.

After electrophoresis, the gels were washed for 1 h in 2% Triton X-100 and incubated in zymography activation buffer (50 mmol/L Tris-HCl, 5 mmol/L CaCl_2_, pH 7.4) for 48 h. Proteolytic activity was evaluated and registered with an ultraviolet light scanner (ChemiDoc Universal Hood, Bio-Rad, Hercules, CA, USA). Gelatinase activity in the samples was analysed in duplicate. Zymographic bands were identified and quantified using ImageJ software (National Institutes of Health, Bethesda, MD, USA).

### 2.2. In Situ Zymography of Resin–Dentine Interfaces

One-millimetre-thick slabs of middle/deep dentine were obtained from extracted human primary or permanent molars (*n* = 3 per group) using the low-speed microtome (Micromet, Remet, Bologna, Italy) with water-cooling. Each slab was sectioned into two pieces; the latter were randomly assigned to two subgroups. The same dentine tissue was used for examining both mineralised dentine and etched dentine: a standardised smear layer on each dentine surface using 600-grit silicon–carbide paper and water lubrication. Half of the samples were left mineralised, whereas the other half were etched with 35% H_3_PO_4_ (etching gel, 3M ESPE, St. Paul, MN, USA) for 15 s and thoroughly rinsed. The etching procedure was performed by one experienced operator. The slabs were subsequently sectioned vertically into two 1 mm thick sticks. Each stick was glued to a microscope slide and polished ~50 µm thick. 

In situ zymography was performed using the procedures reported by Mazzoni et al. [13,14]. Briefly, the self-quenched fluorescein-conjugated gelatine mixture (E-12055; Molecular Probes, Eugene, OR, USA) was placed over the polished dentine surfaces and protected with a coverslip. The samples were incubated overnight at 37 °C in a humid chamber, avoiding direct contact with water or exposure to light. Confocal laser scanning microscopy (Model A1-R, Nikon, Tokyo, Japan) was used to examine the samples after incubation (excitation/emission wavelengths 488/530 nm). The sample examination was performed by one blinded expert operator. Images were taken in 3 different areas of each stick (middle and two outer tooth areas). Each image was made as a stack of images 1 µm apart from each other into the depth of the sample, with the aim of identifying the depth of hydrolysis of the quenched fluorescein-conjugated gelatine substrate. Hydrolysis was presented as green fluorescence. The integrated density of the fluorescence signals was measured using ImageJ software and statistically analysed. The differences in fluorescence intensity between the primary and permanent dentine groups were used as a relative indicator of the differences in the endogenous dentinal enzymatic activity between the tested groups.

### 2.3. Statistical Analysis

The in situ zymography data did not violate the normality (Shapiro–Wilk test) and homoscedasticity assumptions (modified Levene test) required for parametric statistical analysis; therefore, a two-way analysis of variance was used to examine the effect of dentine substrate (primary vs. permanent dentine) and acid-etching (mineralised vs. demineralised dentine), and the interaction of these two factors on the intensity of the fluorescence signals (mean ± SD). The Holm–Šidák statistic was used for post hoc pairwise comparisons. Statistical significance was pre-set at α = 0.05 for all analyses.

## 3. Results

### 3.1. Gelatine Zymography

**Figure 1:**
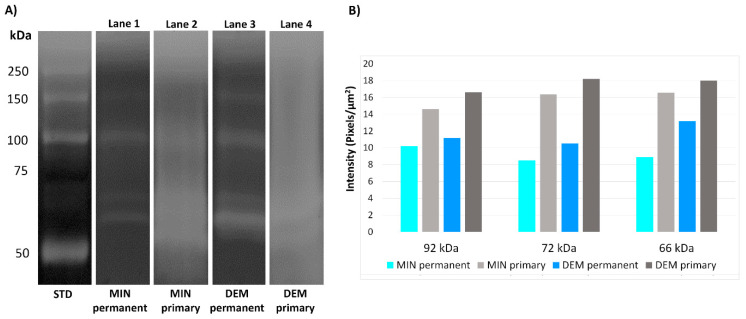
Gelatine zymography of protein extracts from primary and permanent dentine powder: (**A**) electrophoresis of the protein extracts; (**B**) quantification of the density of the MMP bands. STD—standard; MIN—mineralised; DEM—demineralised.

Results of the gelatine zymography and densitometry of the bands created after electrophoresis are shown in Figure 1.

Proteins extracted from the mineralised permanent dentine (G1) contained the pro-form and active form of MMP-2 (72 and 66 kDa, respectively) and the pro-form of MMP-9 (92 kDa), (Figure 1, lane 1). Proteins extracted from the demineralised permanent dentine (G3) showed increased expressions of MMP-2 pro- and active forms and pro-MMP-9 (Figure 1, lane 3). Proteins extracted from the mineralised and demineralised primary dentine (G2 and G4, respectively) showed diffuse, poorly defined bands in the zymograms that vaguely corresponded to the pro-form and active form of MMP-2 and the pro-form of MMP-9 (Figure 1, lane 2 and lane 4, respectively). More well-defined bands were present in the zymograms of the permanent dentine powder. Control zymograms incubated with 5 mM EDTA or 2 mM 1,10-phenanthroline (both being MMP inhibitors) exhibited no enzymatic activity (data not shown).

### 3.2. In Situ Zymography

**Figure 2:**
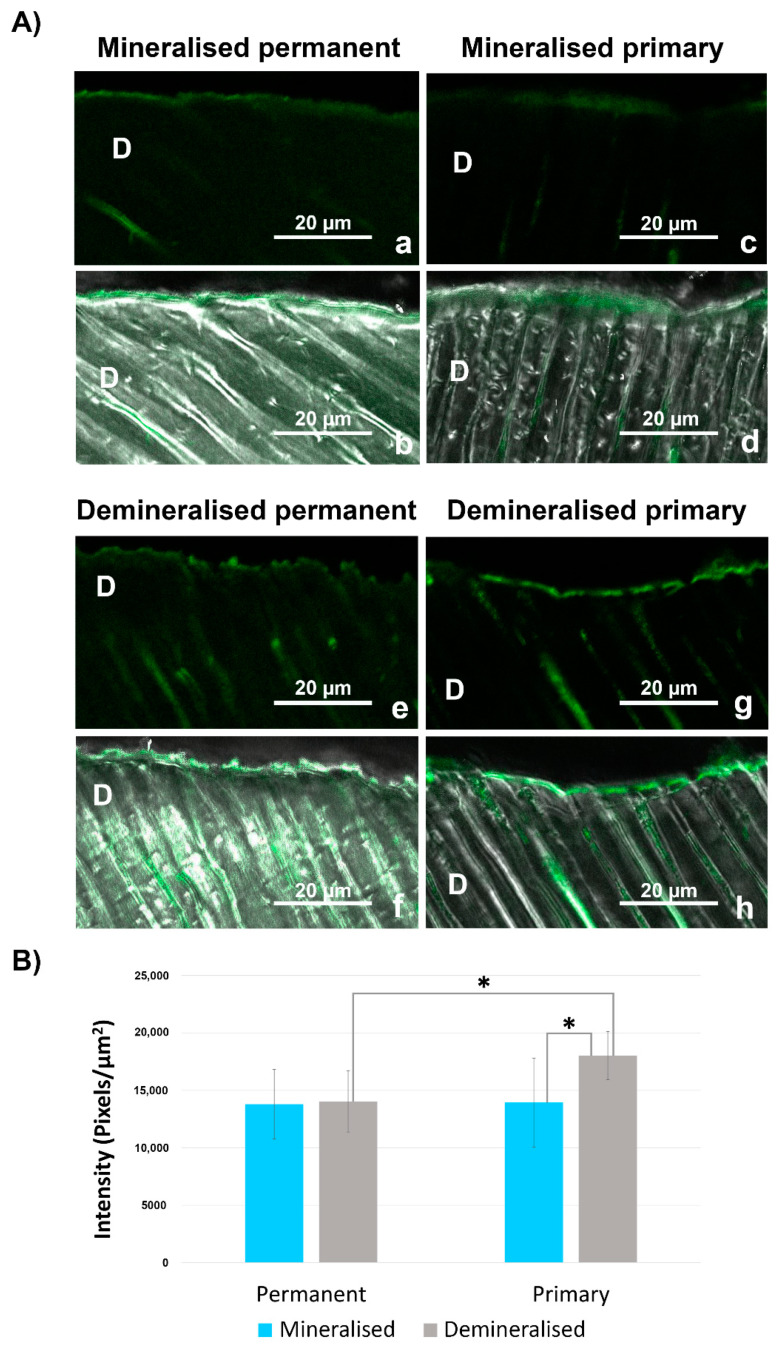
In situ zymography of dentine slices: (**A**) confocal laser scanning microscopy images acquired using the green-channel, showing fluorescence in mineralised and acid-etched permanent and primary dentine (**a**,**c**,**e**,**g**); Confocal laser scanning microscopy images of different groups obtained by merging differential interference contrast images (showing the optical density of dentine) and images acquired with the green channel of the same groups (**b**,**d**,**f**,**h**); (**B**) in situ zymography quantification (mean ± standard deviation). Statistically significantly different groups are marked with an asterisk. Asterisk marks groups that are statistically significantly different from each other (*p* < 0.05). D—dentine.

Differences in in situ dentine enzymatic activity were identified between the investigated groups (Figure 2). The ANOVA demonstrated that both main variables (dentine and etching) as well as their interaction significantly influenced the fluorescent signal density (*p* = 0.004, *p* = 0.003, *p* = 0.007, respectively). For pairwise comparisons of primary vs. permanent dentine, differences were identified between the two substrates only after demineralisation (*p* = 0.001). For pairwise comparisons of mineralised vs. demineralised dentine, differences were identified in primary dentine (*p* = 0.001) but not in permanent dentine (*p* = 0.791) (Figure 2B, mean ± SD).

In situ zymography appeared to be suitable for examining both primary and permanent dentine. Stronger signals were present in the demineralised samples compared with the mineralised samples. In the mineralised samples, gelatinolytic activity was predominantly located on the dentine surface in the form of a thicker layer. Conversely, in demineralised primary and permanent dentine, the dentinal tubules were rendered patent by etching, and gelatinolytic activity was also evident within the dentinal tubules.

## 4. Discussion

Both experimental techniques used in the present study demonstrated differences in the gelatinolytic activity between the two substrates. This warranted rejection of the null hypothesis. However, the gelatine zymography protocol developed for permanent dentine does not seem to be suitable for the examination of primary dentine powder. Conversely, the in situ zymography protocols of thin dentine sections are equally reliable for examining the endogenous enzymatic activities of both primary and permanent dentine. 

The influence of etching and adhesive procedures on the endogenous enzymatic activity in permanent dentine have been successfully tested by means of gelatine zymography and in situ zymography [12,13,14]. Each experimental method has its advantages and disadvantages. Gelatine zymography enables direct identification of the presence of pro-forms and active forms of MMP-2 and MMP-9 in a dentine substrate, the activities of which can be quantified for comparison [12,15,16]. In situ zymography, on the other hand, cannot differentiate between different gelatinolytic enzymes. When this technique is used, it is not possible to determine which of the enzymes is responsible for the degradation of the artificial gelatine substrate that is placed over the polished dentine section [13,14]. However, the advantage of in situ zymography compared to gelatine zymography is that it enables visual identification of the distribution of dentinal endogenous enzymatic activity in different parts of dentinal tissue and the hybrid layer. Similar to gelatine zymography, the activity shown by in situ zymography can be quantified and analysed statistically.

Although several gelatinolytic enzymes are present in permanent as well as primary dentine, our research focused on the gelatinases MMP-2 and -9, because they are the most abundant MMPs in dentine [10,11] and their expression at the level of the molecular weights between 66 and 92 kDa has previously been demonstrated using Western blotting and gelatine zymography [12]. Other enzymes, such as cysteine cathepsin K and cysteine cathepsin B (molecular weights 25 and 38 kDa, respectively), should be further investigated, and the immunological identity of their enzymatic activities should be confirmed by means of Western blotting. The results of gelatine zymography in the present study in the mineralised group of primary dentine were not clearly delineated, with wide bands present in the regions designated as MMP-2 and MMP-9. These bands were even less well-delineated in the demineralised primary dentine, and the activity could not be attributed to the specific MMPs with absolute certainty. This may be due to the differences in the mineral content between permanent and primary teeth. Primary teeth are smaller in size, with thinner enamel and lower levels of Ca and P ions in the enamel [3,4], as well as a lower Ca content and Ca:P weight ratio in dentine [5]. In the investigation of the microstructure of primary and permanent dentine, it was noted that primary dentine contained a higher number of dentinal tubules that are wider in diameter, and hence, less intertubular dentine [6]. Furthermore, the direction of the tubules in permanent dentine was S-shaped, whereas in deciduous dentine, it was predominantly straight, possibly due to the differences between these tissues in the relation to the dentine surface near the dentin–enamel junction and one near the pulp [17]. Deciduous dentine also contains giant tubules, which are uncommon for permanent dentine [18]. Due to the aforementioned compositional, morphological and histological reasons, acid-etching is more easily accomplished in primary dentine [19]. It is speculated that the organic components of the demineralised dentine matrix, which include the MMPs, may be denatured and inactivated by prolonged exposure to phosphoric acid [20]. Hence, a modification of the demineralisation protocol should be considered when performing gelatine zymography on primary dentine powder. Furthermore, in view of the concern that over-etching may adversely affect the clinical success of paediatric resin composite restorations, several authors have advocated the use of short etching times when bonding to primary dentine [21,22,23]. For example, Scheffel et al. recommended a 5 s etching time for primary dentine and at least 10 s for permanent dentine [22]. 

The results of in situ zymography in the present study showed significantly more pronounced enzymatic activity in etched primary dentine, when compared to permanent dentine or mineralised primary dentine. It has been shown that acid-etching can increase MMP expression in dentine [14,24]. Unlike gelatine zymography, in situ zymography generated clear and consistent results in primary dentine. Gelatinolytic activity in primary dentine was morphologically similar and spatially distributed in a similar manner to that observed in permanent dentine. Due to differences in methodology, one cannot directly compare the results of the present study with available literature. Nevertheless, our results appear to be in line with those reported by Osorio et al. [25], in that there is a higher level of MMP-mediated collagen degradation in demineralised primary dentine, compared to demineralised permanent dentine. Furthermore, a recent study also revealed higher quantities of cross-linked carboxyterminal telopeptide of type I collagen (ICTP) and C-terminal crosslinked telopeptide of type I collagen (CTX), greater reduction in elastic modulus and higher dry mass loss in primary compared to permanent dentine [8]. These differences might be due to differences in the composition or functionality of the components of the organic matrix. The extracellular organic matrix of dentine is composed of collagen (90%) and non-collagenous proteins (10%) [10]. Among the non-collagenous proteins, enzymes such as MMPs and cysteine cathepsins play important roles in tooth formation and other physiological processes, as well as pathological processes in tooth tissues, such as caries or hybrid layer degradation [26,27,28,29]. In primary dentine, MMP-9 is involved in tooth resorption [30], whereas this physiologic function is not required in permanent teeth. Hence, it is possible that the MMPs exhibit different functions and levels of activity in primary and permanent dentine, which might have implications in everyday clinical practice. It has been reported that endogenous dentine MMP expression and activation contribute to degradation of the hybrid layer in permanent dentine [31,32,33,34,35]. It is not uncommon to over-etch when using etch-and-rinse adhesives, creating an etching depth that is beyond the depth of infiltration of adhesive resin monomers into the demineralised collagen matrix. The layer of water-filled denuded collagen fibrils at the base of the hybrid layer provides the optimal conditions for degradation of the collagen fibrils by MMPs [10]. In fact, the survival of dental restorations appears to be lower in primary teeth compared to permanent teeth [36,37]. The increase in MMP activity in primary dentine may be a possible reason for lower bond strength in deciduous dentine [25]. 

Further studies are required, using different adhesive systems, to confirm the value of in situ zymography for testing endogenous enzymatic activity in primary dentine. Furthermore, the influence of MMP inhibitors and cross-linkers on bond strength in primary dentine should be investigated.

## 5. Conclusions

Within the limitations of the present study, it may be concluded that primary dentine possesses stronger gelatinolytic activity after acid-etching. This may be related to the differences in morphology and composition between these two dentine substrates. Compared with gelatine zymography of dentine powder, in situ zymography appears to be a more appropriate laboratory technique for investigating endogenous gelatinolytic activity in primary teeth.

## Data Availability

The data presented in this study are available on request from the corresponding author.
